# Predicting antigenic variants of H1N1 influenza virus based on epidemics and pandemics using a stacking model

**DOI:** 10.1371/journal.pone.0207777

**Published:** 2018-12-21

**Authors:** Rui Yin, Viet Hung Tran, Xinrui Zhou, Jie Zheng, Chee Keong Kwoh

**Affiliations:** 1 School of Computer Science and Engineering, Nanyang Technological University, Singapore, Singapore; 2 School of Information and Communication Technology, Hanoi University of Science and Technology, Hanoi, Vietnam; 3 Genome Institute of Singapore, A*STAR, Biopolis, Singapore, Singapore; St. Jude Children’s Research Hospital, UNITED STATES

## Abstract

H1N1 is the earliest emerging subtype of influenza A viruses with available genomic sequences, has caused several pandemics and seasonal epidemics, resulting in millions of deaths and enormous economic losses. Timely determination of new antigenic variants is crucial for the vaccine selection and flu prevention. In this study, we chronologically divided the H1N1 strains into several periods in terms of the epidemics and pandemics. Computational models have been constructed to predict antigenic variants based on epidemic and pandemic periods. By sequence analysis, we demonstrated the diverse mutation patterns of HA1 protein on different periods and that an individual model built upon each period can not represent the variations of H1N1 virus. A stacking model was established for the prediction of antigenic variants, combining all the variation patterns across periods, which would help assess a new influenza strain’s antigenicity. Three different feature extraction methods, i.e. residue-based, regional band-based and epitope region-based, were applied on the stacking model to verify its feasibility and robustness. The results showed the capability of determining antigenic variants prediction with accuracy as high as 0.908 which performed better than any of the single models. The prediction performance using the stacking model indicates clear distinctions of mutation patterns and antigenicity between epidemic and pandemic strains. It would also facilitate rapid determination of antigenic variants and influenza surveillance.

## Introduction

Influenza is an infectious disease that poses significant threat to public health worldwide, especially H1N1 of influenza A virus, which caused several pandemics in history, e.g. the 1918 Spanish flu, leading to millions of deaths [1]. Except for the pandemics, epidemics also cause about 250,00 to 500,000 deaths per year around the world [2]. Hemagglutinin (HA) and neuraminidase (NA) are the most important proteins that characterize influenza A viruses [3]. HA is responsible for binding the virus to host cells with sialic acid on the membranes [4] and NA functions as a tetramer that cleaves sialic acid from cells and virion glycoproteins to prevent clumping of released viruses [5]. However, the accumulation of antigenic shift or drift within HA proteins results in new strains of virus which can not be inhibited effectively by antibodies originally targeted at previous strains and thereby causes new epidemics or pandemics. HA protein cleaves into two chains, namely, HA1 and HA2. HA1 mutates much more frequently than HA2 and is subject to strong selection for novel variations [6]. It is the main objective of this paper to study and predict the antigenic variants to facilitate vaccine recommendation.

Hemagglutinin inhibition (HI) assay is the primary method to determine the antigenicity of influenza viruses and quantitative antibody titers for vaccine selection [7]. However, HI assay is a labour-intensive and time-consuming method, which prompts the development of computational techniques for the prediction of antigenic similarity between antisera and antigens to identify the antigenic variants. The phylogenetic trees combined with antigenic cartography remains prevailing in antigenic analysis. Smith et al. constructed an antigenic map to determine the antigenic evolution of influenza A H3N2 virus from 1968 to 2003 [8]. Lorusso et al. used antigenic cartography to analyze the antigenic properties of 2008 H1 viruses and demonstrated that the viruses in the different phylogenetic clusters are also antigenically divergent [9]. The antigenic patterns and evolution of human influenza A (H1N1) viruses were investigated by Liu et al., who inferred the antigenic clusters from a large-scale sequence data covering the whole epidemic history of H1N1 [10]. Bedford et al. and Du et al. constructed the maps of the global circulation patterns of seasonal flu strains and antigenic evolution, respectively [11, 12]. These previous works depicted the evolutionary paths of influenza and provided the foundation for computational models of antigenicity prediction. Sequence-based methods and imputation-based methods [13] are the most common methods for antigenic prediction. For example, Ren et al. applied random forest regression and support vector regression to identify antigenicity-associated sites in the hemagglutinin protein of A/H1N1 seasonal influenza virus [14]. Yin et al. detected the potential critical virulent sites in past pandemic strains using rule-based methods [15]. Besides, one of the most crucial factors for the success of influenza vaccination is the timely determination of emerging influenza virus antigenic variants. Sun et al. provided a novel, experimentally validated, computational method for determining influenza virus antigenicity based on HA sequences [16]. Yao et al. proposed a joint random forest method for predicting influenza H3N2 antigenicity from hemagglutinin sequence data [17]. Qiu et al. incorporated the structural context of HA protein to calculate the antigenicity for influenza virus A/H3N2 with an accuracy of 0.875 [18]. By building a universal model for all HA subtypes of influenza A viruses based on conserved antigenic structures, Peng et al. achieved an accuracy of 0.77 for predicting antigenic variants of avian influenza H9N2 viruses [19]. Furthermore, Richard Neher et al. showed the antigenic differences measured by serological data are well described by antigenic changes along the path connecting viruses in phylogenetic trees [20]. It allows predicting antigenicity from HA sequences by mapping on the trees. Luksza and Laessig developed a fitness model for haemagglutinin that predicts the evolution of the viral population, which maps the adaptive history of influenza A and suggests guidance for vaccine selection [21].

Despite the availability of these computational methods in identifying antigenicity-associated sites and predicting antigenic variants for influenza, most of the models were built for influenza A/H3N2. There is still insufficient knowledge on the influenza H1N1 that caused numerous epidemics all over the world. Besides, high-performance model for rapid prediction of H1N1 antigenicity from sequences is needed. In this study, we built a stacking model to include all influenza periods of H1N1 based on epidemics and pandemics for the prediction of antigenic variants. To the best of our knowledge, this is the first attempt to take epidemic and pandemic events into account for inferring the antigenic relation between H1N1 virus strains. We categorized influenza strains into two types, pandemic-based and epidemic-based, denoted as “PDM” and “EPD”. The period of five pandemics in recent centuries was shown in Table 1 and the rest of the time is regarded as an epidemic period. In this way, we not only classified the antigenic relation of two influenza strains within a certain period, but also compared antigenic relation of two strains more broadly. By analyzing sequence data and calculating the entropy of each residue position of HA1 protein for strains, we demonstrated that influenza H1N1 went through different variations across the periods. Pearson Correlation Coefficient (PCC) [22] was performed to further identify the distinct mutation patterns of H1N1 strains in each period. Individual prediction models of a pair of strains across periods were constructed for the antigenic variants prediction. Finally, a stacking model was built to predict antigenic variants combining H1N1 epidemic and pandemic strains from all periods. The accuracy of the stacking model exceeded those of above-mentioned for H1N1. Three different feature generation methods applied to the construction of models proved its feasibility and reliability. We believe this model could help to analyze epidemic and pandemic strains. The highlighted mutation patterns and constructed models may also help facilitate rough determination of antigenic variants, the surveillance of influenza and as a reference to the selection of vaccine strains.

**Table 1:** The classification of H1N1 periods and data collection based on chronological pandemics (PDM) and epidemics (EPD).

Event	Type	Year	Number of sequences	Period
1918Spanish	PDM	1918-1920	3	1
SeasonalFlu1	EPD	1921-1976	107	2
1977Russia	PDM	1977-1980	28	3
SeasonalFlu2	EPD	1981-2008	2247	4
2009Swine	PDM	2009-2011	5099	5
SeasonalFlu3	EPD	2012-2016	2208	6

## Materials and methods

### Data collection

We used two types of data in this study, including antigenic data and sequence data. Antigenic data was based on hemagglutination inhibition (HI) assay and collected from diverse sources, such as World Health Organization (WHO), European Centre for Disease Prevention and Control (ECDC), The Francis Crick Institute (FCI), U.S Food and Drug Administration (FDA) and relevant literature. In total, 1772 pairs of HI assay data of HA1 viral strains were obtained for influenza H1N1. As for the sequence dataset of HA1, it was obtained from Influenza Virus Resource (IVR) [23] and Global Initiative on Sharing All Influenza Data (GISAID) [24] on 31 Dec, 2016. The sequence collection was filtered by minimal length 327 (HA1 length) and human host. The dataset comprises 9859 sequences in total after removing duplicate sequences for H1N1. Because hemagglutination inhibition assay was developed in 1940s as the method for quantifying the relative concentration of viruses, bacteria or antibodies [25], no antigenic data is available and there are only scarce sequence data for period 1. Thus we only consider period 2 to 6 in subsequent analysis.

### Data cleaning and preprocessing

We adopted Archetti-Horsfall distance [26] to define the antigenic relations between strains as follows:

$$\begin{array}{c} D_{ij}=\sqrt{\frac{H_{ii}*H_{jj}}{H_{ij}*H_{ji}}} \end{array}$$ (1)

*D*_*ij*_ stands for the antigenic distance, *H*_*ij*_ is the HI titer of strain *i* related to antisera raised against strain *j*. When the value of *D*_*ij*_ is greater than or equal to 4 (a threshold defined by Liao et al. [27]), the antigenic relation between the two strains is considered to be distinct, otherwise it is similar. If the HI titer of the same pair was measured in multiple independent institutions, the median titer value was taken [28]. After removing duplicate pairs, we obtained 937 antigenically distinct pairs and 636 antigenically similar pairs in total.

MAFFT [29] was applied to the collected sequences for multiple sequence alignment. Because of different lengths of HA proteins of H1N1, the aligned sequences showed many insertions and deletions. Thus we only kept the HA1 residues and deleted others including signal peptide in each strain. Moverover, the sequences with a gap ratio greater than 10% were also removed by manual check. The remaining samples comprised 107 strains, 28 strains, 2247 strains, 5099 strains and 2208 strains for periods 2 to 6 respectively shown in Table 1. The antigenic and sequence data of each period can be found in S1 and S2 Files after cleaning and preprocessing.

### Feature engineering

According to our analysis, HA proteins of H1N1 in different periods were probably subject to distinct mechanisms generating antigenic variations at the site level. To build a computational model for the prediction of antigenic variants, three different feature engineering methods were applied to the construction of model built on epidemics and pandemics to test its feasibility and universality, namely, residue-based, ten regional band-based and five epitope region-based methods. Residue-based method uses all the sites on HA1 protein to generate features. By extracting features on single sites between strains, 327 different features were obtained for every pair of strains, where 0 represents the same animo acid between strains on the site and 1 otherwise. The regional bands, defined based on Lees et al. [30], were acquired by the calculation of distance on the C*α* atoms between residues on the top of HA1. Here we adopted ten regional bands to generate new features based on the sites of each band. The number of amino acid changes in each regional band between a pair of sequences was extracted as features. Similarly, epitope regions of H1 [31] to which that the human immune system primarily responds was another way to generate new features in the five epitope regions.

### Model construction

As we know that the influenza strains in different periods may lead to various levels of disease such as epidemics or pandemics. We first investigated the distinct mutation patterns of strains from different periods regardless of whether they were caused by antigenic drift or antigenic shift. We took the distinctions as facts, focusing on highlighting the variation patterns across periods. To validate that the strains in different periods went through distinct antigenic variations, the mutation patterns of HA1 proteins were analyzed by calculating the moving average position information entropy of amino acid sites on HA1, which could reflect the variant patterns of influenza virus antigens. Then, Pearson Correlation Coefficient (PCC) analysis was performed on the information entropy of amino acid positions between periods in terms of moving average position information entropy. These analyses suggested that the H1N1 viruses in different periods have most likely experienced diverse mutation variations. Therefore, we first built models on each type to predict its antigenicity, described as a single model, illustrating potential evolutionary patterns from periods 2 to 6 in the following way.

Set H1N1 strains in periods 2 to 6 be designated by letters A, B, C, D, E. We need select two different strains as a pair. Since the evolution of influenza viruses is in a forward path [32], the strains could evolve within one period to another period chronologically. For example, influenza strains in period 2 could only evolve directly or indirectly into strains in any period from 2 to 6, but not to period 1. Here we didn’t consider the situation that the subtype H1N1 developed into other subtypes. In this case, we built the model for the prediction of antigenic variants based on period 2 by the settings that for a pair of strains, one of the strains was from period 2 and the other strain was from any of the potential forward periods, which could represent antigenic relation of a pair of strains from period 2 and another. This situation is stated as Type II (AA, AB, AC, AD, AE). In this way, Other types of pairs of strains were presented as Type III (BB, BC, BD, BE), Type IV (CC, CD, CE), Type V (DD, DE) and Type VI (EE). We finally obtained 99, 180, 843, 369 and 74 samples from Type II to VI for the training and testing of antigenic relationship between pairs of strains. Single models were first applied to the individual types for the prediction of antigenic variants by several classifiers including logistic regression (LR), support vector machine (SVM), naïve bayes (NB), neural network (NN) and k-nearest neighbour (KNN). Due to the imbalanced distribution of antigenic similar and distinct samples, stratified sampling technique was applied to the training process to construct the validation model. We divided the samples into minor and major classes for each type and randomly selected 80% of each class for training and kept the rest for testing. It would make sure that the samples on each class were selected for training with a ratio of 0.8. This could overcome the overfitting problem that the minor class was overwhelmed by the major class with high accuracy. Three feature generation methods were applied to predict the antigenic relationship between strains for each type. The average accuracy was calculated over 10 runs by these classifiers above.

**Fig 1:**
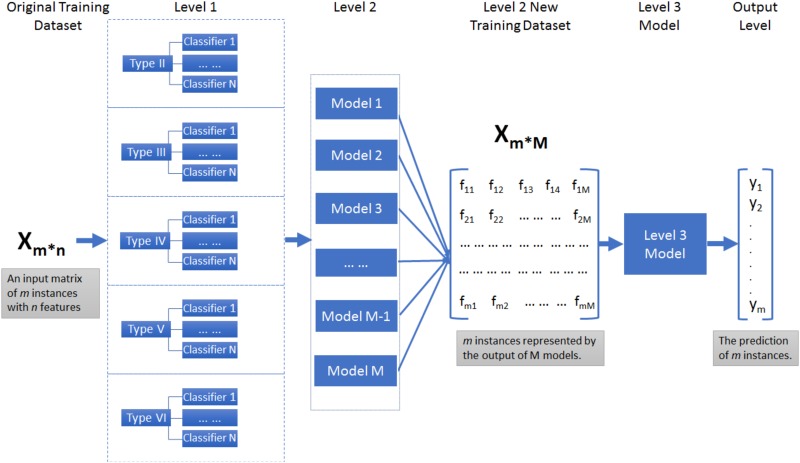
The workflow of stacking model for the antigenic variants prediction based on pandemics and epidemics.

Furthermore, our objective is to provide a universal model for the prediction of antigenic variants of influenza H1N1 that can be applied to all types, so a stacking model was introduced [33]. The stacking model uses a similar idea as *k*-fold cross validation to create out-of-sample predictions that works for small or medium sized datasets. It constructs a predictive model by combining different models as illustrated in Fig 1. In this work, we randomly selected 80% samples containing each type from the original dataset *X*_*m*×*n*_, which contained *m* samples and *n* features. The rest 20% data was used to test on the stacking model. The parameter *n* would change in the training dataset *X*_*m*×*n*_ according to different feature vectors. At level 1, different classifiers were applied on *X*_*m*×*n*_ and tested on the testing data of each type to select the best algorithms as base classifiers in term of their prediction performance. Correspondingly, logistic regression, naïve bayes and neural network were selected to construct the models at level 2 in terms of the better performance on single models. In addition, random forest and gradient boosting were added to construct the models at level 2. These models at level 2 can provide predictions for the outcome of all data, which were then casted into the second level of training data presented as *X*_*m*×*M*_. The parameter *M* stands for the number of new features of the dataset. The model at level 3 was constructed by logistic regression classifier, trained and tested on *X*_*m*×*M*_ to produce the final outcomes of the predictions of antigenic variants. The performance of the stacking model will be compared with the base classifiers. Meanwhile, to study the influence of data bias of different types on the stacking model, we also investigated the stacking model with the model built on balanced datasets. We set 74, the number of Type VI samples, as benchmark number and randomly selected balanced samples from other types. Totally, 370 samples were extracted as new training and testing dataset to predict antigenic relationship between strains. The performance of stacking model with all datasets and balanced datasets was compared to investigate the influence of imbalanced dataset distribution for prediction. Furthermore, ROC (Receiver Operating Characteristic) curve was plotted by the stacking model constructed above to validate its strengthen and stability.

We used the software R version 3.4.0 to conduct all the statistical analyses in this work, including entropy calculation, feature engineering, model construction and validation. The classifiers applied in training and testing models were performed by the package H2O ensemble [34]. The package ROCR was used to plot the ROC curve and calculate the area under ROC curve (AUC) [35].

## Results

**Fig 2:**
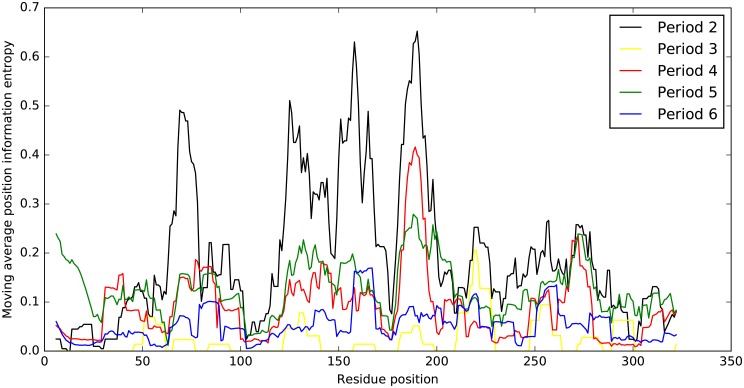
Position-dependent entropy. Moving average position information entropy was calculated with a window size of 11 for HA1 protein if influenza A virus in each period, that is period 2 (black), period 3 (yellow), period 4 (red), period 5 (green) and period 6 (blue). The amino acid position are in H1 numbering system [37].

The HA1 viral protein, as the main antigen of influenza viruses, is the immunodominant part of HA segment [36]. The analysis of mutation patterns of HA1 proteins in different periods was implemented by the calculation of information entropy in each residue position. Because of the conserved sites in aligned sequences of HA1, the value of information entropy would be 0 that caused steep distribution values on different sites when plotted in the figure. Therefore, we used the moving average position information entropy with the window size of 11 to reflect variation patterns of HA1 proteins. The results in Fig 2 presented a diverse patterns of entropy variations of the HA1 residues. For example, the peaks of the curve in period 2 located at site 156 and 190, while the same position in period 3 or 4 was in the valley. Some other similar situations could also be found in the figure such as site 75, 145, 160, 200 and 275. These sites could have undergone diverse mutation patterns in different periods. Although some sites reflected similar mutational tendency in all periods, like site 103 and 210, the overall variation patterns tended to be distinct in different periods. However, the accumulation of these mutation sites in distinct variation patterns could lead to different extent of influenza outbreaks through years.

The correlation analysis between residues’ information entropy on HA1 proteins in different periods further verified distinct mutation patterns shown in Table 2. The correlations of residues’ entropy variations of HA1 protein ranged from 0.03 to 0.69 among periods, which was measured by Pearson Correlation Coefficient. These results indicated a medium (0.3-0.8) or low (0-0.3) correlations of mutation patterns between periods. Especially, the correlation coefficients of period 3-4 and 3-5 were quite low, only attaining 0.05 and 0.03 respectively. It may infer the influenza H1N1 strains appeared in 1977 Russia flu yielded drastic and frequent mutations during the years from 1978 to 2008, when there were intermittent epidemics occurred all over the world. However, compared with strains in period 3, the strains in period 4 are less disparate than those in period 5 that the correlation reached 0.67. It also reasonably accounted for the low correlation of strains between period 3 and period 5. In combination, the results above suggested the diversity of mutation patterns of influenza H1N1 in terms of pandemic and epidemic events, and gave a better insight for understanding the mutation patterns for H1N1 strains.

**Table 2:** Pearson correlation coefficient between different periods (P2 to P6) based on pandemics and epidemics.

H1N1	P2	P3	P4	P5	P6
P2	1.00	0.18	0.69	0.58	0.50
P3	0.18	1.00	0.05	0.03	0.25
P4	0.69	0.05	1.00	0.67	0.36
P5	0.58	0.03	0.67	1.00	0.36
P6	0.50	0.25	0.36	0.36	1.00

Our analysis suggested that HA1 proteins of influenza H1N1 in different periods probably experienced distinct variation patterns. The single models, trained and tested in each type, were constructed by several basic classifiers. These classifiers consisted of logistic regression (LR), support vector machine (SVM), naïve bayes (NB), neural network (NN) and k-nearest neighbour (KNN). The predicting accuracy of single models on average was shown in Table 3 by three feature generation methods. Logistic regression, neural network and naïve bayes classifiers were selected for the construction of stacking model according to the superior experimental results shown in red. Furthermore, we also explored whether the single model in one type could perform well applied to others. We only presented the results with best classifiers, that is, logistic regression, neural network and naïve bayes, corresponding to residue-based, regional band-based and epitope region-based features respectively, to test the performance of all types. The model performed quite well when tested within the same type that was highlighted in bold but relatively poor on other types in most cases shown in Table 4. For example, the single model on Type III achieved an accuracy over 0.9 on average based on three different feature generation methods, but it only obtained the accuracy ranging from 0.3 to 0.8 for others. Similar cases can be found in other single models, which further validated the relative insufficient performance to predict the antigenic variants for mutation patterns of different types using single models.

**Table 3:** Single model prediction accuracy on residue-based, regional band-based and epitope region-based features within the same type.

Classifier	Feature generation methods		
	Residue-based	Regional band-based	Epitope region-based
LR	0.943	0.883	0.809
SVM	0.851	0.855	0.820
NB	0.532	0.737	0.823
NN	0.907	0.919	0.804
KNN	0.848	0.784	0.817

**Table 4:** Single model performance on residue-based, regional band-based and epitope region-based computational models trained and tested in Type II-VI. “Acc”,accuracy; “Sen”, sensitivity; “Spe”, specificity.

Training	Testing	Residue-based			Regional band-based			Epitope region-based		
		Acc	Sen	Spe	Acc	Sen	Spe	Acc	Sen	Spe
Type II	II	**0.949**	**0.5**	**0.989**	**0.949**	**0.875**	**0.956**	**0.888**	**0.375**	**0.934**
	III	0.533	0.857	0.506	0.9	0.077	0.969	0.783	0	0.849
	IV	0.692	0.559	0.743	0.498	0.081	0.659	0.610	0.423	0.682
	V	0.722	0.758	0.375	0.25	0.134	0.892	0.472	0.458	0.553
	VI	0.689	0.672	0.75	0.391	0.327	0.625	0.459	0.465	0.437
Type III	II	0.666	0.25	0.703	0.626	0.375	0.648	0.878	0	0.956
	III	**0.95**	**0.5**	**0.987**	**0.938**	**0.785**	**0.951**	**0.861**	**1**	**0.849**
	IV	0.710	0.329	0.856	0.615	0.273	0.746	0.698	0.517	0.768
	V	0.336	0.288	0.607	0.576	0.583	0.535	0.777	0.846	0.392
	VI	0.702	0.810	0.312	0.445	0.344	0.812	0.837	0.931	0.5
Type IV	II	0.282	0.75	0.241	0.747	0.125	0.802	0.858	0	0.934
	III	0.455	0.714	0.433	0.894	0.428	0.933	0.85	1	0.837
	IV	**0.891**	**0.782**	**0.934**	**0.792**	**0.303**	**0.980**	**0.703**	**0.739**	**0.689**
	V	0.548	0.532	0.642	0.491	0.458	0.678	0.793	0.913	0.125
	VI	0.581	0.568	0.625	0.5	0.396	0.875	0.851	0.931	0.312
Type V	II	0.575	0.75	0.560	0.434	1	0.384	0.414	1	0.362
	III	0.761	0.714	0.765	0.827	0.928	0.819	0.322	1	0.265
	IV	0.581	0.850	0.478	0.442	0.944	0.25	0.317	0.965	0.0761
	V	**0.937**	**0.967**	**0.767**	**0.934**	**0.971**	**0.732**	**0.810**	**0.935**	**0.107**
	VI	0.918	0.948	0.815	0.770	0.810	0.625	0.851	1	0.312
Type VI	II	0.394	0.75	0.362	0.686	0.25	0.725	0.868	0	0.945
	III	0.167	1	0.097	0.827	0.194	0.714	0.855	0.928	0.849
	IV	0.550	0.623	0.521	0.432	0.589	0.371	0.666	0.717	0.646
	V	0.793	0.910	0.142	0.578	0.637	0.25	0.793	0.913	0.125
	VI	**0.986**	**0.982**	**1**	**0.945**	**0.982**	**0.812**	**0.851**	**1**	**0.312**

We further studied how the partition of different periods of HA1 proteins made the impact on the model of antigenic variants prediction. Due to the rapid antigenic shift or drift that brings about the occurrence of antigenic variants, the effects on antigenicity of specific mutations have not been clear painted. The changes of antigenicity depend not only on the property and number of the amino acid substitutions but also on the amino acids currently encoded at certain key positions in HA1 [38]. The performance of the models based on different feature vectors didn’t show much difference, which indicated that the generation of new features from residue sites was probably not the main factor in influencing the prediction results. Some more elements, such as environment and individual immune system, need to be considered for the possibility of H1N1 variation mechanism that distinguishes epidemics and pandemics, which is out of scope in this paper.

**Fig 3:**
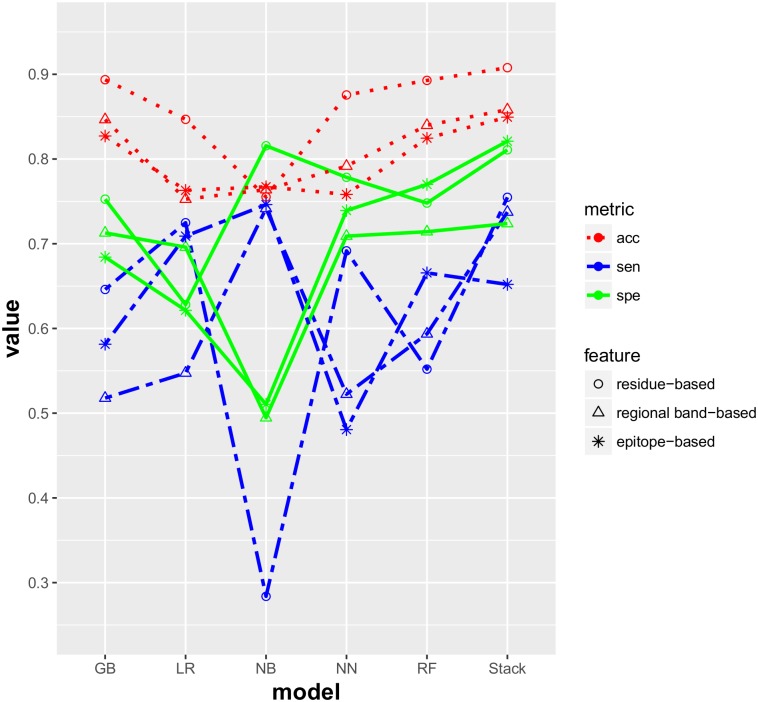
Performance comparison of residue-based, regional band-based and epitope-based computational models trained and tested across different types. “acc”: accuracy; “sen”: sensitivity; “spe”: specificity.

The pilots above prompt the construction of a comprehensive model that can contain all different types for antigenicity prediction. Therefore, a stacking model was built to predict the antigenic variants of HA1 proteins of influenza H1N1 for all circumstances. Logistic regression, neural network and naïve bayes were selected as base classifiers to constitute the models at level 1 of stacking model due to their performance in single models. Except for these three classifiers, we also introduced two ensemble classifiers, random forest (RF) and gradient boosting (GB), to jointly form the models at level 2, adding diversity and strengthening the robustness. The stacking model at level 3 was built by logistic regression because of the small dimension for new training dataset obtained from level 2. The performance of the stacking model was presented in Fig 3 in comparison with other classifiers used at the level 2 for the prediction of antigenic variants by three different feature vectors. We calculated the mean value of each type obtained by these models in terms of accuracy, sensitivity and specificity. The details of the results could be found in S3 File. Accordingly, the best predicting accuracy was 0.908, achieved by stacking model with residue-based features. The sensitivity and specificity of the stacking model also displayed competitiveness, which was 0.755 and 0.811 respectively. We may infer that the more residues we used, the better performance it would achieve on the antigenic variants prediction. Although the results based on five epitope regions and ten regional bands performed not as well as residue-based method, we observed that the stacking model still slightly outperformed other models.

**Fig 4:**
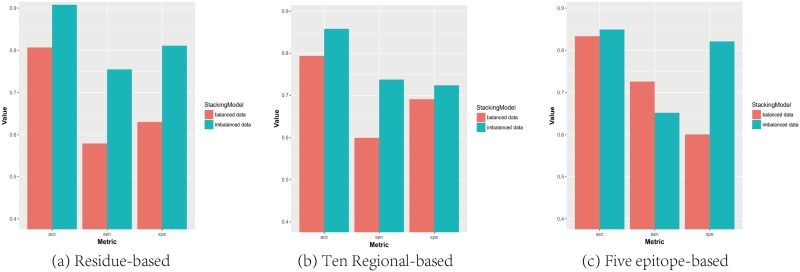
The performance of stacking model with imbalanced and balanced datasets based on three feature generation methods. “acc”: accuracy; “sen”: sensitivity; “spe”: specificity. (a) The performance of residue-based stacking model (b) The performance of ten regional-based stacking model (c) The performance of five epitope-based stacking model.

**Fig 5:**
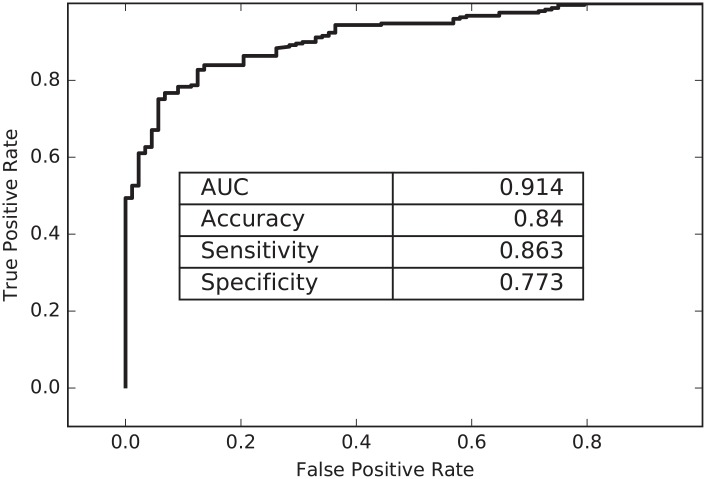
The Receiver Operating Characteristic (ROC) curve of the stacking model predicting the antigenic variants of influenza A H1N1 virus.

In comparison, we also investigated and compared the performance of the stacking model with imbalanced and balanced datasets. The results in Fig 4 only showed the average performance containing all types. (Details can be seen in S3 File) It suggested that the stacking model with imbalanced datasets comprehensively presented slight better performances over balanced datasets by all three different feature generation methods except for the sensitivity on five epitope-based method. It was implausible that the evenly distributed datasets negatively contributed to the performance of the model. This was probably due to the small-scale balanced samples we extracted for training and testing, which was not a lot to learn for classifiers. Indeed, the insufficient antigenic data in different periods would set obstacles on the performance of the model. Even though, our stacking model has successfully predicted antigenic relationship between strains based on limited data. Moreover, Fig 5 showed the ROC (Receiver Operating Characteristic) curve with a significant proportion of area under curve (AUC) of 0.915 by the stacking model. The AUC value illustrated the matching degree with the experiment data ranging from 0 to 1 and the larger of the value, the better of the matching level. These outcomes demonstrate that the stacking model built upon epidemics and pandemics not only performs better than models using other classifiers in the experiments, but also achieves comprehensive improvement compared with the results of single models in Table 4.

## Discussion

Since the occurrence of 1918 Spanish pandemic, the influenza H1N1 has been evolving and circulating up to now. The rapid mutations of the antigenicity of influenza A virus are unceasingly causing other epidemics or pandemics that severely threaten public health. In this work, we analyzed and compared the mutation patterns of HA1 protein from six periods defined by the characterization of epidemics and pandemics. The moving average position information entropy and Pearson Correlation Coefficient were applied on mutation patterns analysis. Due to the scarce data of strains in period 1, the analysis of mutation patterns of period 1 was excluded. The results in Fig 2 showed similarity of mutation patterns in some regions of the residue sites, for example, the residue sites from 180 to 200 indicated the same variation trend across periods. But the overall variation trend indicates the distinct patterns of epidemics or pandemics in different periods, which is in accordance with update of influenza vaccines every year [39–41]. Besides, the value of moving entropy information of period 3 is small compared with other periods. This could be caused by insufficient sequences collected for the calculation of entropy information.

The purpose of applying three different methods of feature generation for antigenic variants prediction is to validate the feasibility and reliability of the model constructed based on epidemics and pandemics. The residue-based method converts the number of amino acids changes in each residue site between a pair of sequences as one feature and all the sites are taken for generating feature vectors. Although not all sites are closely correlated with antigenic variation, we obtain impressive performance in the prediction of antigenic relation between strains. The features extracted from five epitope regions focus on the regions that encompass the sites at which antibodies bind to HA1 [42]. Different from residue-based method, epitope region-based method would more directly reflect the relation between antigenic sites and variants. Moreover, some researchers regard that the evolutionary selective pressure has varied over time on some specific amino acid position [43], indicating the significance of detecting other important sites influencing immune response. Therefore, ten regional bands have been proposed [30] and the features generated from ten regional bands not only contain many residues in epitope regions but also implicate potential crucial sites that locate outside five antigenic epitopes. Fig 3 suggests that the model using residue-based features achieves better prediction results than regional band-based and epitope regional-based features. We might infer that the more features applied for the prediction of antigenic variants, the better performance will be.

However, the relative few antigenic data is available in some periods for H1N1 viruses, which could hinder the development of computational models and further hamper the performance of prediction on antigenic variants. Meanwhile, the mutation pattern analyses demonstrated the diversity of influenza virus antigens in different periods. Single model was built to predict their antigenicity at first. The results turned out that these single models performed much worse trained and tested in different types than in the same type, suggesting single model is not capable of predicting antigenicity across types with acceptable confidence. Therefore, a stacking model was developed that integrated all the situations of antigenic variants prediction of influenza H1N1 HA1 proteins in different types using all the antigenic data. Feature vectors extracted by three different methods were applied on the stacking model. Although it performed slightly inferior to single models which were trained and tested in the same type in Table 3, the performance of predicting antigenic variants by the stacking model across types was much better than the single model. Besides, we can also find that the stacking model showed the best performance compared with other classifiers applied at level 2, exceeding 0.87 in accuracy on average. This could be the optimized classifiers applied individual types that constitute the level 2 of the base models, enabling us to average out the noise from diverse models and thereby enhance the generalized prediction results. This is sometimes referred as an approach named “wisdom of crowds”, pulling from the age-old philosophy of Aristotle [44]. By combining antigenic data from all types in terms of epidemic and pandemic information and using diverse modeling approaches, the stacking model gain more accuracy and robustness than a fine-tuned single model can obtain.

Even if we could obtain good performance on the prediction results based on different influenza epidemics and pandemics, there are still some space on the improvement of specificity and sensitivity. For example, the sensitivity is low in some of the constructed models, especially for the prediction of Type II. (See in S3 File) One crucial factor is the limited antigenic data we can collect and the imbalanced classes of similar and distinct pair of strains. We may bring in penalty mechanism or assign unequal weight distribution on samples in the training process to tune the bias towards the minority class. Nevertheless, this stacking model validates its feasibility and reliability on the prediction of antigenic variants of H1N1 influenza A virus. The analysis also raises the perspective of how to select a proper model when predicting the antigenic variants for influenza viruses in different types with few antigenic data available. The model based on the chronological evolutionary paths of H1N1 that caused epidemics and pandemics with closest possible genetic relations could provide a suitable choice to target antigenic variants. Meanwhile, the stacking model built on diverse epidemic and pandemic periods of antigenic data would capture more comprehensive mechanisms behind antigenic variation. Our future work moves towards the improvement of these models and the identification of potential virulent sites that can distinguish the formation of epidemics and pandemics caused by influenza H1N1 viruses.

## Conclusion

In conclusion, we divide the influenza strains of H1N1 epidemic and pandemic events into different periods chronologically. Mutation pattern analysis of HA1 of influenza A H1N1 proves that the amino acid changes and antigenic variation of strain pairs differ across periods. The single prediction models constructed show clear poorer performance when tested in the antigenic relation with other types. Therefore, the construction of the stacking model of prediction antigenic variants of influenza H1N1 overcomes the challenge of diverse mutation variations, combining all the situations. Residue-based, five epitope region-based and ten regional band-based feature vectors applied in the training process prove the feasibility and reliability of the stacking model built on chronological epidemic and pandemic periods by achieving a good performance on the prediction of antigenic variants. This study not only paves a path on the study of distinct antigenic evolution of influenza H1N1 virus, but also gives insight on the potential mutation sites that distinguish past epidemic and pandemic outbreaks. It also provides a new perspective for the antigenic variants prediction with reliability and accelerates the selection of vaccine strains.

## Supporting information

S1 File Antigenic and corresponding sequence data of each type for calculating the antigenic relation between strains of influenza H1N1 HA proteins. (CSV)

S2 File HA Sequence data for the analysis of mutation patterns of epidemic and pandemic strains in each period. (FAS)

S3 File The performance comparison between stacking model and other models and comparison between stacking model with imbalanced and balanced datasets by residue-based, ten regional band-based and five epitope region-based methods. (XLSX)

## References

[1] Johnson NP, Mueller J. Updating the accounts: global mortality of the 1918-1920 “Spanish” influenza pandemic. Bulletin of the History of Medicine. 2002;76(1):105–115. 10.1353/bhm.2002.0022 11875246

[2] World Health Organization. Fact sheet No 211: Influenza (Seasonal). WHO: Geneva, Switzerland, April. 2009;.

[3] de Wit E, Fouchier RAM. Emerging influenza. Journal of clinical virology: the official publication of the Pan American Society for Clinical Virology. 2008;41 1:1–6. 10.1016/j.jcv.2007.10.017 18340670 PMC2768345

[4] Russell RJ, Kerry PS, Stevens DJ, Steinhauer DA, Martin SR, Gamblin SJ, et al Structure of influenza hemagglutinin in complex with an inhibitor of membrane fusion. Proceedings of the National Academy of Sciences. 2008;105(46):17736–17741. 10.1073/pnas.0807142105 PMC2584702 19004788

[5] Brown E. Influenza virus genetics. Biomedicine & Pharmacotherapy. 2000;54(4):196–209. 10.1016/S0753-3322(00)89026-5 10872718

[6] Plotkin JB, Dushoff J. Codon bias and frequency-dependent selection on the hemagglutinin epitopes of influenza A virus. Proceedings of the National Academy of Sciences. 2003;100(12):7152–7157. 10.1073/pnas.1132114100 PMC165845 12748378

[7] De Jong J, Palache A, Beyer W, Rimmelzwaan G, Boon A, Osterhaus A. Haemagglutination-inhibiting antibody to influenza virus. Developments in biologicals. 2003;115:63–73. 15088777

[8] Smith DJ, Lapedes AS, de Jong JC, Bestebroer TM, Rimmelzwaan GF, Osterhaus AD, et al Mapping the antigenic and genetic evolution of influenza virus. science. 2004;305(5682):371–376. 10.1126/science.1097211 15218094

[9] Lorusso A, Vincent AL, Harland ML, Alt D, Bayles DO, Swenson SL, et al Genetic and antigenic characterization of H1 influenza viruses from United States swine from 2008. Journal of General Virology. 2011;92(4):919–930. 10.1099/vir.0.027557-0 21177926 PMC3133703

[10] Liu M, Zhao X, Hua S, Du X, Peng Y, Li X, et al Antigenic Patterns and Evolution of the Human Influenza A (H1N1) Virus. Scientific reports. 2015;5. 10.1038/srep14171 PMC4585932 26412348

[11] Bedford T, Riley S, Barr IG, Broor S, Chadha M, Cox NJ, et al Global circulation patterns of seasonal influenza viruses vary with antigenic drift. Nature. 2015;523(7559):217–220. 10.1038/nature14460 26053121 PMC4499780

[12] Du X, Dong L, Lan Y, Peng Y, Wu A, Zhang Y, et al Mapping of H3N2 influenza antigenic evolution in China reveals a strategy for vaccine strain recommendation. Nature communications. 2012;3:709 10.1038/ncomms1710 22426230

[13] Barnett JL, Yang J, Cai Z, Zhang T, Wan XF. AntigenMap 3D: an online antigenic cartography resource. Bioinformatics. 2012;28(9):1292–1293. 10.1093/bioinformatics/bts105 22399675 PMC3338011

[14] Ren X, Li Y, Liu X, Shen X, Gao W, Li J. Computational identification of antigenicity-associated sites in the hemagglutinin protein of a/h1n1 seasonal influenza virus. PloS one. 2015;10(5):e0126742 10.1371/journal.pone.0126742 25978416 PMC4433265

[15] Yin R, Zhou X, Ivan FX, Zheng J, Chow VTK, Kwoh CK. Identification of Potential Critical Virulent Sites Based on Hemagglutinin of Influenza a Virus in Past Pandemic Strains. In: ICBBS’17; 2017.

[16] Sun H, Yang J, Zhang T, Long LP, Jia K, Yang G, et al Using sequence data to infer the antigenicity of influenza virus. MBio. 2013;4(4):e00230–13. 10.1128/mBio.00230-13 23820391 PMC3705446

[17] Yao Y, Li X, Liao B, Huang L, He P, Wang F, et al Predicting influenza antigenicity from Hemagglutintin sequence data based on a joint random forest method. Scientific Reports. 2017;7 10.1038/s41598-017-01699-z PMC5431489 28484283

[18] Qiu J, Qiu T, Yang Y, Wu D, Cao Z. Incorporating structure context of HA protein to improve antigenicity calculation for influenza virus A/H3N2. Scientific reports. 2016;6:31156 10.1038/srep31156 27498613 PMC4976332

[19] Peng Y, Wang D, Wang J, Li K, Tan Z, Shu Y, et al A universal computational model for predicting antigenic variants of influenza A virus based on conserved antigenic structures. Scientific Reports. 2017;7:42051 10.1038/srep42051 28165025 PMC5292743

[20] Neher RA, Bedford T, Daniels RS, Russell CA, Shraiman BI. Prediction, dynamics, and visualization of antigenic phenotypes of seasonal influenza viruses. Proceedings of the National Academy of Sciences. 2016;113(12):E1701–E1709. 10.1073/pnas.1525578113 PMC4812706 26951657

[21] Łuksza M, Lässig M. A predictive fitness model for influenza. Nature. 2014;507(7490):57 10.1038/nature13087 24572367

[22] Benesty J, Chen J, Huang Y, Cohen I. Pearson correlation coefficient In: Noise reduction in speech processing. Springer; 2009 p. 1–4.

[23] Sayers EW, Barrett T, Benson DA, Bolton E, Bryant SH, Canese K, et al Database resources of the national center for biotechnology information. Nucleic acids research. 2012;40(D1):D13–D25. 10.1093/nar/gkr1184 22140104 PMC3245031

[24] Shu Y, McCauley J. GISAID: Global initiative on sharing all influenza data–from vision to reality. Eurosurveillance. 2017;22(13). 10.2807/1560-7917.ES.2017.22.13.30494 PMC5388101 28382917

[25] Hirst GK. The quantitative determination of influenza virus and antibodies by means of red cell agglutination. Journal of Experimental Medicine. 1942;75(1):49–64. 10.1084/jem.75.1.49 19871167 PMC2135212

[26] Ndifon W, Dushoff J, Levin SA. On the use of hemagglutination-inhibition for influenza surveillance: surveillance data are predictive of influenza vaccine effectiveness. Vaccine. 2009;27(18):2447–2452. 10.1016/j.vaccine.2009.02.047 19368786

[27] Liao YC, Lee MS, Ko CY, Hsiung CA. Bioinformatics models for predicting antigenic variants of influenza A/H3N2 virus. Bioinformatics. 2008;24(4):505–512. 10.1093/bioinformatics/btm638 18187440

[28] Zacour M, Ward BJ, Brewer A, Tang P, Boivin G, Li Y, et al Standardization of Hemagglutination Inhibition Assay for Influenza Serology Allows for High Reproducibility between Laboratories. Clinical and Vaccine Immunology. 2016;23(3):236–242. 10.1128/CVI.00613-15 26818953 PMC4783428

[29] Katoh K, Standley DM. MAFFT multiple sequence alignment software version 7: improvements in performance and usability. Molecular biology and evolution. 2013;30(4):772–780. 10.1093/molbev/mst010 23329690 PMC3603318

[30] Lees WD, Moss DS, Shepherd AJ. A computational analysis of the antigenic properties of haemagglutinin in influenza A H3N2. Bioinformatics. 2010;26(11):1403–1408. 10.1093/bioinformatics/btq160 20388627 PMC2913667

[31] Deem MW, Pan K. The epitope regions of H1-subtype influenza A, with application to vaccine efficacy. Protein Engineering, Design & Selection. 2009;22(9):543–546. 10.1093/protein/gzp027 PMC3307478 19578121

[32] Dunham EJ, Dugan VG, Kaser EK, Perkins SE, Brown IH, Holmes EC, et al Different evolutionary trajectories of European avian-like and classical swine H1N1 influenza A viruses. Journal of virology. 2009;83(11):5485–5494. 10.1128/JVI.02565-08 19297491 PMC2681951

[33] Polikar R. Ensemble based systems in decision making. IEEE Circuits and systems magazine. 2006;6(3):21–45. 10.1109/MCAS.2006.1688199

[34] LeDell E. h2oEnsemble: H2O ensemble learning. R package version 01. 2016;8.

[35] Robin X, Turck N, Hainard A, Tiberti N, Lisacek F, Sanchez JC, et al pROC: an open-source package for R and S+ to analyze and compare ROC curves. BMC bioinformatics. 2011;12(1):77 10.1186/1471-2105-12-77 21414208 PMC3068975

[36] Russell R, Gamblin S, Haire L, Stevens D, Xiao B, Ha Y, et al H1 and H7 influenza haemagglutinin structures extend a structural classification of haemagglutinin subtypes. Virology. 2004;325(2):287–296. 10.1016/j.virol.2004.04.040 15246268

[37] Burke DF, Smith DJ. A recommended numbering scheme for influenza A HA subtypes. PloS one. 2014;9(11):e112302 10.1371/journal.pone.0112302 25391151 PMC4229193

[38] Bush RM, Bender CA, Subbarao K, Cox NJ, Fitch WM. Predicting the evolution of human influenza A. Science. 1999;286(5446):1921–1925. 10.1126/science.286.5446.1921 10583948

[39] Centers for Disease Control and Prevention. Updated interim influenza vaccination recommendations–2004-05 influenza season. MMWR Morbidity and mortality weekly report. 2004;53(50):1183 15614237

[40] Fiore AE, Shay DK, Broder K, Iskander JK, Uyeki TM, Mootrey G, et al Prevention and control of influenza: recommendations of the Advisory Committee on Immunization Practices (ACIP), 2008. MMWR Recommendations and reports: Morbidity and mortality weekly report Recommendations and reports. 2008;57(RR-7):1–60. 18685555

[41] Centers for Disease Control and Prevention. Prevention and control of influenza with vaccines: recommendations of the Advisory Committee on Immunization Practices (ACIP)–United States, 2012-13 influenza season. MMWR Morbidity and mortality weekly report. 2012;61(32):613 22895385

[42] Wilson IA, Cox NJ. Structural basis of immune recognition of influenza virus hemagglutinin. Annual review of immunology. 1990;8(1):737–787. 10.1146/annurev.iy.08.040190.003513 2188678

[43] Blackburne BP, Hay AJ, Goldstein RA. Changing selective pressure during antigenic changes in human influenza H3. PLoS pathogens. 2008;4(5):e1000058 10.1371/journal.ppat.1000058 18451985 PMC2323114

[44] Güneş F, Wolfinger R, Tan PY. Stacked Ensemble Models for Improved Prediction Accuracy;.

